# Effects of mindfulness-based intervention on adolescents emotional disorders: A protocol for systematic review and meta-analysis

**DOI:** 10.1097/MD.0000000000028295

**Published:** 2021-12-23

**Authors:** Dan-Feng Tang, Li-Qiong Mo, Xin-Chu Zhou, Jun-Hong Shu, Lei Wu, Dong Wang, Fei Dai

**Affiliations:** a Institute of Physical Education, Wuchang Institute of Technology, Wuhan, China; b Institute of Physical Education, Wuhan University of Arts and Science, Wuhan, China.

**Keywords:** adolescents, emotional disorders, meta-analysis, mindfulness, systematic review

## Abstract

**Background:**

The vulnerability of adolescents to emotional disorders such as stress, anxiety, anger, depression, and emotional breakdown is a matter of great concern and urgent need. Studies in several countries and regions have reported higher prevalence of depression, stress, and anxiety in adolescents. Several studies have shown that mindfulness-based interventions have an ameliorative effect on both emotional disorders and psychological problems in adolescents. The purpose of this study is to systematically analyze the effects of mindfulness-based intervention on emotional disorders and psychological problems in adolescents, and to provide a reasonable mindfulness-based intervention program for adolescents with emotional disorders.

**Methods:**

Electronic databases including Google Scholar, EMBASE, Web of Science, PubMed, the CNKI, the Chinese Science and Technology Periodical Database, VIP, Wanfang, and Cochrane Library. These databases will be searched to identify randomized controlled trials (RCTs) published before October 2021. Only Chinese and English literature will be included. We will use the criteria provided in Cochrane Handbook 5.3.0 for quality assessment and risk assessment and Revman 5.3 software for meta-analysis. The primary outcome are mainly evaluated by PHCSS, SDS and SAS in adolescents.

**Conclusion:**

The results of this study may provide a strong basis for improving emotional disorders and psychological problems in adolescents.

Systematic review registration: INPLASY2021110054.

## 1 Introduction

The issue of adolescent mental health and emotional disorders has received increasing attention in recent years. The World Health Organization (WHO) states that worldwide, 20% of adolescents (ages 10–19), in any given year, experience mental health problems, most commonly depression or anxiety disorders. These psychological problems and emotional disorders can affect not only the adolescent's school and life,^[1–3]^ but even substance abuse and suicidal tendencies.^[4–6]^ There is growing evidence that adolescents with dysthymic disorders have difficulty regulating their emotions and may experience a range of problems such as low self-esteem, fear, sadness, and social impairment.^[7–8]^ As a result, the WHO's priorities for advancing the field of adolescent mental health in 2012 include improving adolescents’ coping skills and adapting creative, complementary treatment models that can be implemented in low-resource settings.

There is growing evidence that adolescents’ inability to regulate their emotions due to psychological vulnerability, the presence of low self-esteem and emotional disorders are the main causes of various psychological disorders. Studies have reported a dramatic increase in the prevalence of emotional disorders in the adolescent population, and studies have reported emotional intelligence and internalization problems, depression and anxiety problems, and substance abuse problems that can be caused by emotional disorders, which will also become a major problem affecting adolescent development.^[9–10]^ Because of the many causes that trigger psychological disorders in adolescents, in addition to a range of therapeutic approaches such as biomedical interventions, electrotherapy and medication, some mindfulness practice such as yoga have gained widespread attention as interventions to improve mental health and deal with emotional disorders.^[11–14]^ Therefore, mindfulness practice have a positive impact on emotional disorders and psychological problems in adolescents and should be used as an adjunctive treatment to medication.

## 2 Methods

### 2.1 Study registration

This review protocol is registered with the International Platform for Registration of Systematic Review and Meta-Analysis Protocols under registration number INPLASY2021110054 (https://inplasy.com/inplasy-2021-11-0054/). This systematic review protocol will be conducted and reported in strict accordance with the Preferred Reporting Items for Systematic Reviews and Meta-Analyses (PRISMA)^[15]^ statement guidelines.

### 2.2 Inclusion criteria for study selection

#### 2.2.1 Type of studies

Published randomized controlled trials (RCTs) of the effects of mindfulness practice on emotional disorders in adolescents reported in Chinese or English.

#### 2.2.2 Types of participants

1. Adolescents (10–18 years old) with a diagnosis of mood disorder.
2. No limitation on duration of illness, gender, or race.
3. No organic brain injury or other neuropsychiatric disorders.
4. No medications affecting the neurological or psychiatric system.
5. Have not participated in other clinical trials in the last 3 months.

#### 2.2.3 Types of interventions

##### 2.2.3.1 Experimental interventions

Mindfulness practice, including yoga, tai chi, qigong, and meditation.

##### 2.2.3.2 Control interventions

Electrotherapy, medication, or conventional treatment.

#### 2.2.4 Types of outcome measures

Changes in self-awareness, depression, and anxiety in adolescents.

### 2.3 Exclusion criteria

1. Duplicate publications.
2. Protocols, conferences, abstracts, non-full text
3. Animal experiments
4. Non-RCTs
5. Systematic review and meta-analysis.

### 2.4 Data sources

For literature on mindfulness practice for adolescent emotional disorders published before October 2021, search databases will include Google Scholar, EMBASE, Web of Science, PubMed, the CNKI, the Chinese Science and Technology Periodical Database, VIP, Wanfang, and Cochrane Library.

### 2.5 Searching strategy

The search terms will include “yoga” or “taichi” or “taijiquan” or “Taiji boxing” or “ tai chi” “qigong” or “health qigong ”or “meditation” or “mindfulness practice,” and “mood disorders” or “emotional disorders” or “anxiety” or “depression” or “dysthymic disorders” or “childhood neurosis” and “youth” or “teens” or “adolescents” or “teenagers.”

### 2.6 Literature screening and data extraction

In literature screening, 2 researchers first independently read the titles and abstracts of the retrieved literature to determine inclusion or exclusion, and then further review the full text for in-depth screening. In case of disagreement, a third reviewer will participate in the literature counting screening process. Excel 2016 will be used to record data related to the eligible literature, including:

1. Study characteristics: title, author's name, publication, year of publication, and availability of follow-up.
2. Participants: gender, age, and duration of disease.
3. Intervention: type of mindfulness practice, frequency of exercises, and duration of each exercise, etc.

**Figure 1:**
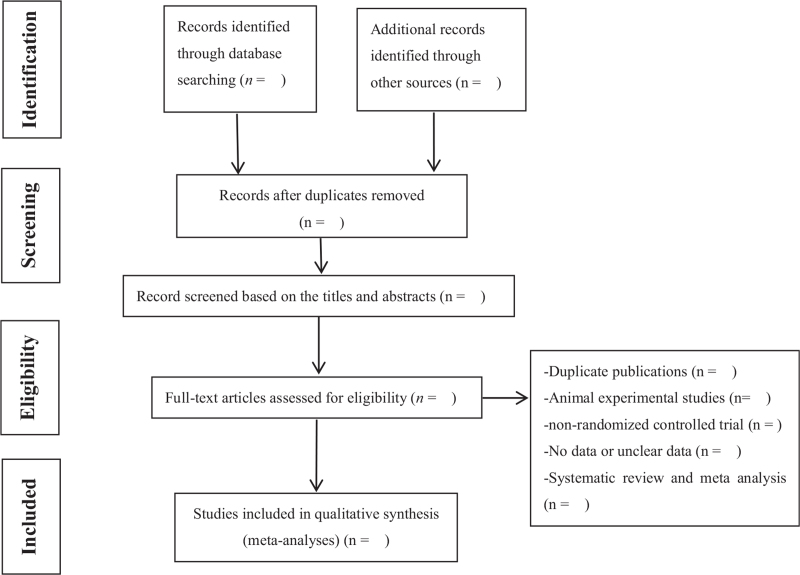
Study selection flow diagram.

The details of the specific screening will be shown in the PRISMA flowchart^[15]^ (Fig. 1).

### 2.7 Assessment of risk of bias

The two authors will evaluate the methodological quality of RCTs according to the Cochrane Handbook for the Evaluation of Intervention Systems, independently assessing the quality of trials included in the evaluation by hidden allocation (selection bias); blinding (performance bias and detection bias); participant blinding (performance bias); outcome assessment blinding (detection bias); completeness of outcome data (attrition bias); selective reporting (reporting bias); and other biases. If any disagreements arise they will be resolved with a third investigator until consensus is reached.

### 2.8 Data analysis

#### 2.8.1 Assessment of heterogeneity

Heterogeneity assessment will be performed by the Review Manager (v.5.3.5). According to the Cochrane Handbook, We will use chi-square tests and *I*^2^ statistics to assess heterogeneity. If low heterogeneity (*P* > .10, *I*^2^ < 50%), a fixed-effects model will be used for the meta-analysis; otherwise, a random-effects model will be used.

#### 2.8.2 Data synthesis

Two authors independently extract the relevant information for each study. A third author will check all data for accuracy. Review Manager 5.3 (Cochrane Collaboration, Oxford, UK) will be used to assess risk of bias, heterogeneity, sensitivity, and subgroup analysis. We will calculate weighted estimates across trials and interpret the results.

#### 2.8.3 Subgroup analyzes

We grouped gender, age, duration of illness, type of mindfulness-based practice, duration and frequency of practice to explore possible reasons for the high heterogeneity.

#### 2.8.4 Sensitivity analysis

Sensitivity analysis will be performed by excluding tests one by one and observing whether there is a significant change in the synthesis results. If significant changes are present, further analysis will be performed and a prudent decision will be made whether to proceed with the synthesis. If there is no significant change, it indicates that the merger can be performed.

### 2.9 Assessment of publication bias

If more than 10 studies are available for analysis, publication bias will be assessed by generating a funnel plot. A symmetrical distribution of data in the funnel plot indicates no publication bias. If it is not symmetrical, we will further analyze the reasons for bias and provide a reasonable explanation.

### 2.10 Evidence evaluation

The Grades of Recommendations Assessment, Development and Evaluation system (GRADE) will be used to assess the quality of our evidence.^[16]^ Evidence quality levels will be classified as high, moderate, low, and very low.

## 3 Discussion

Past investigations have shown that mindfulness-based interventions are effective in improving symptoms of anxiety and depression in both adolescents and adults, making this a promising natural approach to treating emotional disorders.^[17]^ Research has shown promising results for mindfulness-based interventions in alleviating psychological symptoms associated with anxiety and depression in adolescents, and better results for children exhibiting mental health disorders and higher levels of psychological distress.^[18,19]^ Results from RCTs on adolescents suggest that MBI is significantly effective in reducing intrusive thinking, depression, anxiety, stress overload, and aggression, and increases empathy and optimism, while effectively improving emotion regulation skills.^[20–22]^ The breathing and movement modalities of the MBI process can also benefit many other children by teaching adolescents important emotion regulation skills to prevent mental health and stress-related symptoms and improve adolescents’ sense of well-being.^[23–24]^ In recent years, mindfulness-based interventions have been increasingly applied to the treatment and improvement of emotional disorders and psychological problems in adolescents. A large number of scholars have studied the specific effects of mindfulness-based interventions on adolescent emotional disorders from different perspectives, including the common symptoms of depression, anxiety, and fear. In these studies, there may be some variability in the results due to the variability in age, gender, duration, and degree of illness of the participants selected for the mindfulness-based intervention, as well as the specific methods of implementation of the mindfulness-based intervention. Therefore, we will provide reasonable and effective exercise prescriptions to alleviate emotional disorders and psychological problems in adolescents through a meta-analysis of relevant RCTs.

## Author contributions

**Conceptualization:** Danfeng Tang, Fei Dai.

**Data curation:** Li-qiong Mo.

**Formal analysis:** Danfeng Tang, Li-qiong Mo.

**Investigation:** Xin-chu Zhou.

**Methodology:** Xin-chu Zhou, Jun-hong Shu.

**Resources:** Fei Dai.

**Software:** Lei Wu, Dong Wang.

**Supervision:** Fei Dai.

**Validation:** Xin-chu Zhou, Jun-hong Shu.

**Visualization:** Lei Wu, Dong Wang.

**Writing – original draft:** Danfeng Tang.

**Writing – review & editing:** Li-qiong Mo, Fei Dai.
